# Factor VII Deficiency in an End-Stage Renal Disease Patient With Recurrent Thrombosis: A Case Report

**DOI:** 10.7759/cureus.48560

**Published:** 2023-11-09

**Authors:** Ali Shakhshir, Mo’tasem Dweekat, Dalia Hamayel, Omar A Safarini, Johnny Amer, Ahmad Enaya, Sultan Snober

**Affiliations:** 1 Department of Medicine, An-Najah National University Hospital, Nablus, PSE; 2 Internal Medicine, Al-Watani Hospital, Ministry of Health, Nablus, PSE; 3 Department of Internal Medicine, An-Najah National University Hospital, Nablus, PSE; 4 Department of Internships, Palestinian Ministry of Health, Nablus, PSE; 5 Department of Hematology, Faculty of Medicine and Health Sciences, An-Najah National University Hospital, Nablus, PSE; 6 Department of Vascular Surgery, An-Najah National University Hospital, Nablus, PSE

**Keywords:** factor vii deficiency, end-stage renal disease (esrd), shunt dysfunction, high inr, thrombosis

## Abstract

Congenital factor VII deficiency is a rare bleeding disorder with variable presentations. Thromboembolism is a well-established complication of this heterogeneous disease. As it is a rare disease, there is no information regarding its treatment when it is present with other comorbidities such as end-stage renal disease. This study describes a 47-year-old male with multiple comorbidities who was recently diagnosed with end-stage renal disease. He had recurrent admissions to the hospital due to thrombotic arteriovenous access failure as well as acute coronary syndrome, despite a high international normalized ratio that was resistant to replacement therapy. Eventually, apixaban became his main treatment regimen. This case needs to be reported because it is rare in terms of including a factor VII deficiency patient with end-stage renal disease, as well as to emphasize the unclear recommendations available for patients with factor VII deficiency and end-stage renal disease. International collaboration may be the best course of action to study enough patients and come up with effective recommendations.

## Introduction

Coagulation factor VII (FVII) is a plasma vitamin K-dependent protease produced by the liver [1]. The reported prevalence of factor VII deficiency (FVIID) is approximately one symptomatic individual per 500,000 people [2]. However, the true prevalence is likely higher due to the many asymptomatic or minimally symptomatic individuals [2].

Congenital FVIID, most frequent among the rare autosomal inherited bleeding disorders, is a heterogeneous disease with clinical manifestations ranging from asymptomatic to life-threatening hemorrhage [2]. Generally, congenital FVIID is divided into types I and II. Type I patients have a deficiency of both FVII activity and antigen, while type II patients have only a deficiency of FVII activity with variable levels of the antigen [3].

According to studies, a striking feature of this bleeding disorder is that the correlation between circulating FVII and the clinical manifestation is not clear [4]. Interestingly, FVIID does not protect affected patients from venous thromboembolism [2].

To our knowledge, there are no reported cases of FVIID with recurrent thrombotic events. There are also no reported cases of FVIID in end-stage renal disease (ESRD) patients requiring renal replacement therapy. Here, we report the case of a 47-year-old male with ESRD who presented due to recurrent arteriovenous thrombosis of unknown cause (at the time of presentation) and was discovered to have FVIID.

## Case presentation

We present the case of a 47-year-old male with a history significant for hypertension, type 2 diabetes mellitus, ischemic heart disease with percutaneous coronary intervention, heart failure with reduced ejection fraction, and a heavy smoking history of 40 pack-years. His surgical history was significant for an uncomplicated open cholecystectomy.

The patient first presented to our hospital for follow-up after being diagnosed at an outside facility with ESRD. He was undergoing hemodialysis through a left subclavian temporary catheter. The initial plan was to insert a permanent catheter and then create an arteriovenous fistula (AVF) for regular hemodialysis.

On admission, he was found to have a central line-associated infection related to the temporary catheter, which was subsequently treated. Once his infection was controlled, he started preparations for the insertion of a permanent catheter. Coagulation studies showed an international normalized ratio (INR) of 3.3 (reference range: 0.8-1.1), a prothrombin time of 40 seconds (reference range: 11-13.5 seconds), and an activated partial thromboplastin time (aPTT) within the normal range. Due to the risk of excessive bleeding, permanent catheter insertion was postponed, and a jugular temporary dialysis catheter was inserted instead. Interestingly, no excessive bleeding was reported during the procedure.

The patient continued his hemodialysis through the temporary catheter for almost a month, but eventually, he inadvertently removed it. In an attempt to correct the coagulopathy, he received four units of fresh frozen plasma (FFP) with little improvement in his INR. At this point, mixing studies normalized his INR, and he was diagnosed with undetermined factor deficiency. Due to his resistant coagulopathy, he underwent a procedure to insert a right femoral permanent catheter after receiving six units of cryoprecipitate and continued his regular hemodialysis sessions. Two weeks later, the patient presented with painful lower limb swelling and was diagnosed by Doppler ultrasound with a right lower limb deep vein thrombosis related to the tunneled catheter. The catheter was subsequently removed, and a new temporary internal jugular vein catheter was inserted. Testing of multiple coagulation factors revealed an FVII activity level of 6% (normal value more than 70%). Considering he had no evidence of liver disease or vitamin K deficiency and he was not on vitamin K antagonists, he was diagnosed with congenital FVIID.

Subsequently, a right arm brachiocephalic AVF was created after using NovoSeven® Recombinant Factor VIIa. He continued his dialysis sessions through the shunt for four months until he presented with shunt failure. Shuntography showed chronic total occlusion, and shuntoplasty was attempted using 2,500 units of unfractionated heparin but failed to adequately recanalize the shunt. Notably, the INR was 2.9 at the time. He then underwent a femoral permanent catheter insertion to continue his dialysis sessions.

He was then admitted to the hospital for acute coronary syndrome (ACS) and underwent cardiac catheterization. In-stent restenosis was found, and percutaneous transluminal coronary angioplasty with a drug-coated balloon to the left anterior descending (LAD) was performed. Five units of FFP and 2,000 units of unfractionated heparin were used during the procedure, and no complications were reported.

Despite the fact that he claimed strict compliance with dual antiplatelet agents (aspirin and clopidogrel) and an INR of 3.7, the patient was readmitted two months later with another episode of ACS. Recurrence of in-stent restenosis was found on cardiac catheterization, and percutaneous coronary intervention with a drug-eluting stent to the LAD was performed. Notably, no replacement therapy or anticoagulation was used during the procedure. At this point, a right brachio-basilic AVF was performed with no excessive bleeding. Six weeks later, a shunt transposition was performed, and the patient started his regular hemodialysis through it. Four units of FFP were given as replacement therapy four days before and during the transposition procedure.

**Figure 1:**
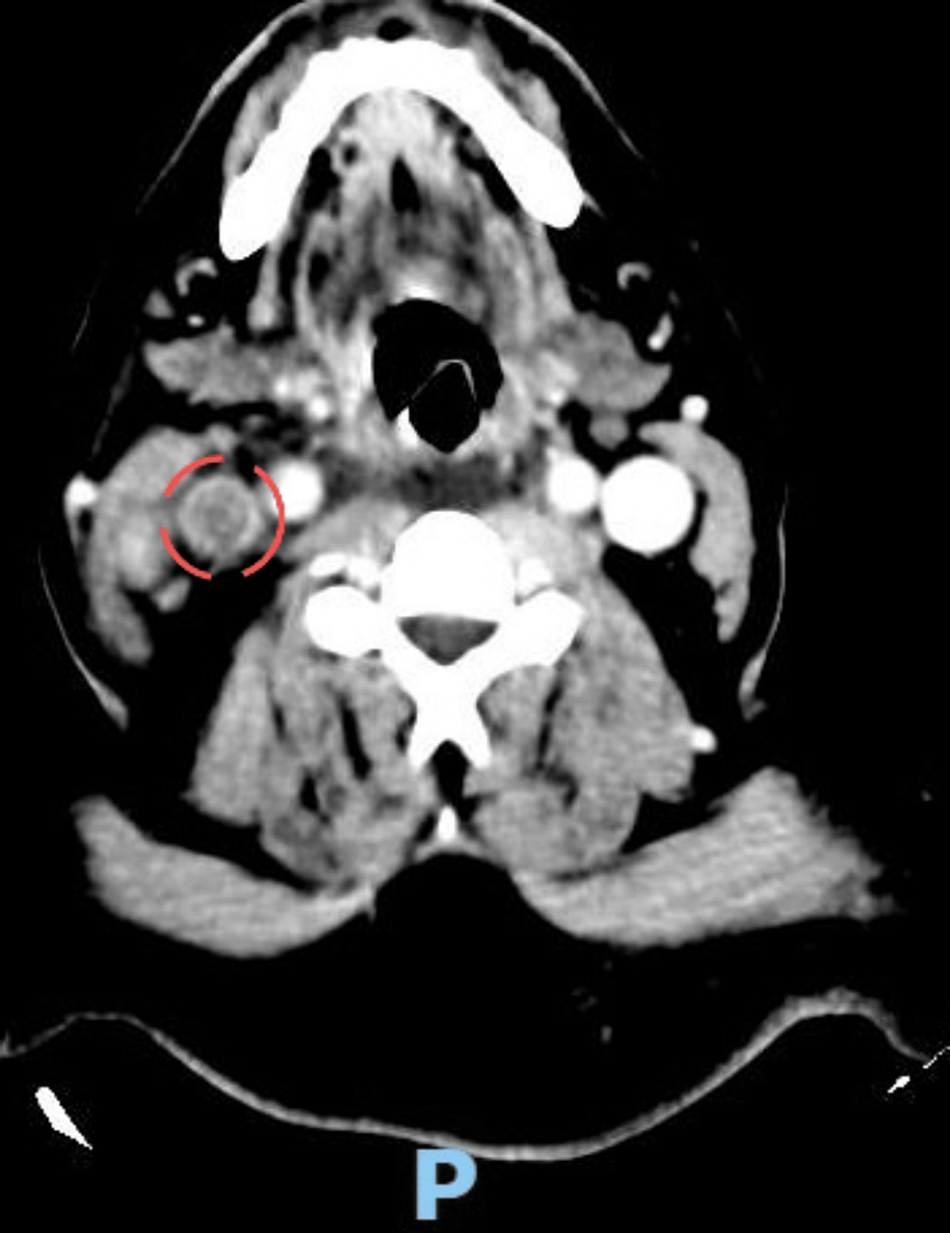
CT venography showing a filling defect of the right internal jugular vein (dashed red circle).

One month later, the patient presented with severe neck pain along with a fever. CT angiography confirmed an internal jugular vein thrombosis without intracranial extension, as shown in Figure 1. A multidisciplinary team discussion reached a consensus to start the patient on renal-adjusted oral apixaban 2.5 mg twice daily.

To date, the patient is on regular hemodialysis through his AVF with no new thrombotic or hemorrhagic episodes.

## Discussion

Coagulation factor deficiencies are known to be mainly hemorrhagic disorders, and FVIID is no exception. Moreover, FVIID is heterogeneous in its bleeding manifestations in that it can cause no bleeding in some patients while leading to severe hemorrhages in others [1]. This heterogeneity is only partially explained by zygosity and the genetic defect [5]. Additionally, plasma FVII activity and bleeding tendency are poorly correlated [4].

Venous and arterial thromboembolism are established complications of coagulation factor deficiency, in general, and FVIID, in particular [6]. A review in 2010 reported that the majority of patients with FVIID who developed thrombosis had prothrombotic risk factors such as surgery, old age, or replacement therapy [7]. Our patient did not report any previous bleeding or thrombosis and had undergone a cholecystectomy before presentation with no complications. Unfortunately, there are no records available to access his preoperative workup or coagulation studies. However, we suspect that the patient developed the manifestations of the disease after the deterioration of his kidney function.

Patients with ESRD on hemodialysis are known to have increased risks of both hemorrhage and thrombosis. Increased bleeding risk is thought to be multifactorial due to the accumulation of medications, including anticoagulants used during hemodialysis, as well as platelet dysfunction. Increased risk for thromboembolism stems from accelerated atherosclerosis and chronic platelet activation from contact with artificial surfaces [8].

To our knowledge, there are no reported cases of FVIID with ESRD developing multiple thromboembolic events. Considering that the patient’s thrombotic events started after developing ESRD, it is possible that the pathophysiologic changes associated with ESRD and hemodialysis contributed to triggering his symptoms of FVIID. Our patient did have multiple risk factors for atherosclerosis and thromboembolism, but the unusual location and timing of those thrombosis events point toward a cause other than atherosclerosis.

Most reported cases of FVIID-causing thrombotic events included either one or two thrombosis events, whereas our patient developed several. Failure of access due to thrombosis is a well-known complication, but our patient had too many failures. Naturally, there are no reported cases of FVIID requiring AVF creation, which, in itself, requires careful consideration and evaluation from the medical community.

Replacement therapy, including FFP, prothrombin complex concentrate, and recombinant activated FVII, is the main therapeutic option for such patients [1]. Unfortunately, only FFP is available at our hospital. Interestingly, INR should be corrected following the administration of FFP, which was not what we experienced [2]. This could be due to the low dose of FFP used, which could not be increased due to the risk of circulatory overload.

Overall, FVIID remains a rare disease, and the literature on anticoagulation in such patients is scanty. Arellano-Rodrigo et al. highlighted the difficulty of finding an effective and safe anticoagulant in their description of two cases [9]. Based on those findings, vitamin K antagonists carry a high risk of both bleeding and thrombosis. Several reports have concluded that heparin (low molecular weight and unfractionated) may be the most suitable anticoagulant in FVIID patients with thrombosis [10]. In our case, unfractionated heparin was used during the cardiac angiography procedure and hospital admission. The response was monitored with aPTT and activated clotting time, with no bleeding episodes.

The major challenge was choosing an effective anticoagulant that is safe for ESRD and FVIID and that can be used for the long term. Although subcutaneous enoxaparin (low-molecular-weight heparin) may appear to be an attractive option, its use in patients with severe renal insufficiency in the therapeutic dose range is associated with increased bleeding risk [11]. Additionally, the anti-factor Xa level is not available in our hospital. Direct oral anticoagulants have become the standard of care for most patients with thromboembolic disorders. Considering its predominantly hepatic clearance, apixaban has been approved for use in venous thromboembolism and ESRD.

This case adds to the existing literature and describes a patient with FVIID who developed ESRD requiring regular hemodialysis with recurrent thrombotic access failure. Moreover, choosing an anticoagulant for a patient with ESRD where a vitamin K antagonist is not an option is challenging. We report this case to emphasize that a multidisciplinary team approach in such cases is crucial for safe management. In addition, more data on thromboembolism in rare factor deficiencies are needed to guide therapy.

## Conclusions

Thromboembolism in ESRD or FVIID, each on its own, is challenging to manage. International collaboration may be the only way to investigate enough patients with FVIID to generate effective recommendations. Surgical interventions in any patient are associated with the risk of thromboembolism. Consequently, protocols for the perioperative management of patients with factor deficiency are necessary.
